# B7-H4: A potential therapeutic target in adenoid cystic carcinoma

**DOI:** 10.18632/oncotarget.28661

**Published:** 2024-11-22

**Authors:** Luana Guimaraes de Sousa, Renata Ferrarotto

**Keywords:** salivary gland cancer, adenoid cystic carcinoma, rare cancer, b7-h4, antibody drug conjugate

Adenoid cystic carcinoma (ACC) is a rare malignancy of secretory glands with dual biological and clinical behavior. Although most patients initially present with locally advanced disease, 50–60% of cases experience distant metastasis, for which there are limited therapeutic options [1]. Our team has previously reported two major molecular ACC subtypes based on proteogenomic and clinical data [2]. ACC-I, the most aggressive type of ACC, is characterized by solid histology, NOTCH1 mutation enrichment, early development of metastasis including to visceral organs and poor prognosis (median overall survival (mOS) = 3.4 ys). While ACC-II is more prevalent (66% of the cases) and characterized by non-solid histology (cribriform and/or tubular), upregulation of p63 and receptor tyrosine kinases, predominantly lung metastasis and an overall indolent course (mOS = 23 ys). These differences underscore the intrinsic heterogeneity of ACC and the need for personalized therapies to improve clinical outcomes of patients facing this disease.

Given the molecular and clinical heterogeneity of ACC, we hypothesized that these two molecular ACC subtypes would also have distinct tumor immune microenvironments (TIME). To that end, we explored the ACC TIME contexture using RNA sequencing (RNA-seq) and spatial proteomics (imaging mass cytometry, (IMC)), and examined its association with molecular subtypes and prognosis [3]. Although both ACC subtypes have a relatively low density of immune cells in the TIME, we paradoxically found that the most aggressive ACC-I had a higher density of immune cells, including cytotoxic T cells (FDR <0.2), which are usually associated with better prognosis in solid cancers. However, our spatial proteomics analysis demonstrated that the immune cells in ACC-I’s TIME are more restricted to the stroma and do not infiltrate the tumor core as much as in ACC-II, reflecting an immune-excluded tumor.

Notably, our RNA-seq analysis, revealed significant upregulation of B7-H4, an inhibitory immune checkpoint, particularly in ACC-I [2]. No other immune checkpoint proteins were expressed at significant levels in ACC. B7-H4 is a type I transmembrane protein predominantly expressed in tumor cells and antigen-presenting cells. It inhibits T cell responses, aids in tumor immune evasion and proliferation, and is emerging as a promising therapeutic target in solid cancers [4]. Given the immune exclusion demonstrated by our spatial analysis, we sought to investigate the role of B7-H4 in the context of immune evasion.

**Figure 1:**
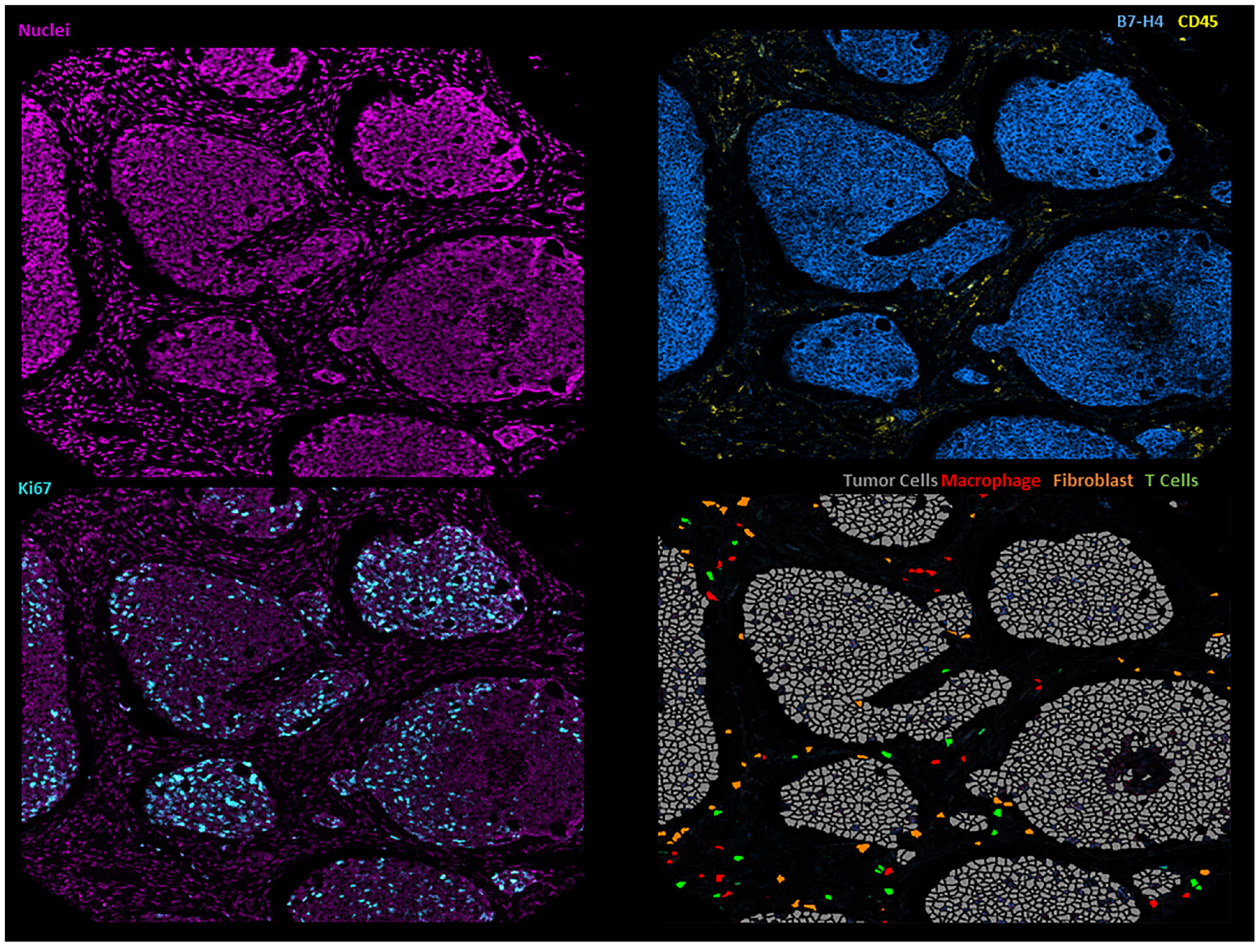
Illustrative imaging mass cytometry images of the ACC microenvironment.

Interestingly, we found that B7-H4 expression was directly associated with T cell restriction to the stroma (*P* = 0.003) and with poor survival (FDR <0.01), even after adjusting for histology and stage. These findings suggest an important role of B7-H4 in the mechanism of immune evasion in ACC-I (Figure 1). This association between B7-H4 and immune evasion is not exclusive to ACC and has been reported in other tumors, such as breast cancer [5, 6].

Given its strong expression in the poor prognosis ACC-I subgroup, we explored the therapeutic potential of B7-H4 pre-clinically using three patient-derived xenograft (PDX) models: two ACC-I models (ACCX9 and ACCX11) with high B7-H4 expression per immunohistochemistry, and one ACC-II model (ACC5M1) with low B7-H4 expression [3]. We screened these models with AZD8205, a novel B7-H4-targeting antibody-drug conjugate (ADC) that employs a topoisomerase 1 inhibitor as its payload. A single dose of AZD8205 significantly inhibited tumor growth and led to regression in 100% (*n* = 30) of the ACC-I/B7-H4 high models, with complete responses in 70 to 90% of ACC-I mice. No treatment effect was observed in the ACC-II/B7-H4-low model. These results provide a robust rationale to investigate B7-H4 as a therapeutic target for B7-H4 expressing ACC.

Based on our findings, a phase I trial with the B7-H4 ADC SGN-B7H4v has opened a cohort to include patients with ACC (NCT05194072). A second trial with another B7-H4 ADC has also started enrolling ACC patients (NCT05377996). These trials represent attractive, rationale therapeutic opportunities for patients facing this rare, aggressive, and chemo-refractory disease, for which no systemic therapy is currently available.
